# Age-stratified analysis reveals heterogeneity in factors associated with gastrointestinal bleeding in IgA vasculitis: a retrospective study in a large volume center

**DOI:** 10.3389/fimmu.2026.1883230

**Published:** 2026-10-21

**Authors:** Jinxi Zhao, Lulu Rao, Ruiheng Xie, Quxin Juhou, Dongqing Wang, Xiaomin Shi, Wei Zhang, Lei Shi, Yan Peng, Xiaohong Wang, Xiaowei Tang

**Affiliations:** 1 Department of Gastroenterology, the Affiliated Hospital of Southwest Medical University, Luzhou, China; 2 Department of Gastroenterology, Xuzhou Central Hospital, Xuzhou Clinical School of Xuzhou Medical University, Xuzhou, China; 3 Department of Endoscopic Medicine, the Affiliated Hospital of Southwest Medical University, Luzhou, China

**Keywords:** age factors, gastrointestinal bleeding, IgA vasculitis, neutrophil-to-albumin ratio, predictive model, systemic immune-inflammation index

## Abstract

**Background:**

Gastrointestinal (GI) bleeding represents a severe complication of IgA vasculitis (IgAV). Most current analyses of risk factors aggregate patients across all ages, potentially obscuring critical age-specific differences. This study aimed to delineate factors independently associated with GI bleeding in IgAV across distinct age groups.

**Methods:**

We conducted a retrospective study involving 964 IgAV patients stratified into four age cohorts: preschool (3–6 years, n=192), school-age (7–12 years, n=313), adolescent (13–17 years, n=202), and adult (>18 years, n=257). Univariable analyses were followed by Firth’s penalized logistic regression within each age cohort to evaluate factors associated with GI bleeding. Model discrimination was evaluated using the area under the receiver operating characteristic curve (AUC), with optimism correction performed via 1,000 bootstrap resamples.

**Results:**

The pattern of factors associated with GI bleeding differed across age strata. In preschool children, NAR was significantly associated with GI bleeding (OR = 5.88). In school-age children, NAR (OR = 2.02), summer onset (OR = 2.47), and medium and high SII (OR = 2.72/3.70) were associated with GI bleeding. In adolescents, high SII (OR = 6.22) and log(FIB/GLO) (OR = 4.57) remained significant, while in adults, high SII was the predominant associated factor (OR = 9.53). These principal associations remained significant after FDR correction for multiple comparisons. The optimism-corrected AUC values of the age-specific models ranged from 0.686 in preschool children to 0.784 in adults.

**Conclusion:**

The factors associated with GI bleeding in IgAV showed heterogeneous patterns across age strata, with neutrophil-related inflammatory markers more prominent in younger children and systemic inflammatory and coagulation-related markers more prominent in older groups. These exploratory findings support further investigation of age-specific risk assessment in IgAV.

## Introduction

IgA vasculitis (IgAV), the most common systemic vasculitis in children, is typically self-limiting but may lead to serious complications. Among these, gastrointestinal (GI) bleeding represents one of the most severe and clinically consequential manifestations, often requiring urgent intervention and contributing substantially to morbidity (1–3). Early identification of patients at increased risk is therefore essential to enable timely management and improve clinical outcomes. Numerous studies have investigated potential risk factors, such as elevated inflammatory markers and coagulation parameters (4–7). However, most existing studies have evaluated IgAV across broad age ranges, frequently combining pediatric and adult populations within a single analytical framework. Such an approach may overlook important age-related differences in disease phenotype, underlying mechanisms, and clinical prognosis (8, 9).

This approach may obscure critical, age-dependent variations in pathophysiology. The immune system, vascular integrity, and hemostatic mechanisms undergo substantial age-dependent maturation from childhood through adulthood, including progressive changes in innate and adaptive immune regulation and coagulation homeostasis (10–12). Given the age-related differences in IgAV phenotype and prognosis, the factors associated with vasculitic injury and bleeding risk may also vary across developmental stages. A uniform risk assessment strategy may therefore be insufficient to capture these age-specific vulnerabilities and could limit the precision of clinical decision-making (9, 12).

Despite this logical premise, a systematic, age-stratified analysis of risk factors for GI bleeding in IgAV is lacking in the current literature. To address this gap, we conducted a retrospective study to characterize the risk profile of IgAV-associated GI bleeding across four predefined age groups: preschool children (3–6 years), school-age children (7–12 years), adolescents (13–17 years), and adults (>18 years). We hypothesized that the pattern of factors associated with GI bleeding might differ across age strata. By delineating these age-specific associations, this study aims to establish a more precise framework for risk stratification, with the potential to facilitate earlier identification of high-risk patients, guide targeted interventions, and ultimately reduce the burden of severe gastrointestinal complications.

## Methods

### Study design and population

This retrospective cohort study was conducted at the Affiliated Hospital of Southwest Medical University and approved by the Institutional Review Board (Approval No. KY2025303). We systematically reviewed the electronic health records of all patients diagnosed with IgA vasculitis (IgAV; Henoch–Schönlein purpura) between January 2019 and December 2024. The diagnosis of IgAV was established according to recognized international and national criteria.

Patients were classified according to the presence of gastrointestinal (GI) bleeding, which was recorded as a binary outcome based on documented overt bleeding manifestations and/or endoscopic evidence when available. A total of 1,115 patients were initially screened, including 484 patients with GI bleeding and 631 patients without GI bleeding. After application of the predefined eligibility criteria, 147 patients from the non-bleeding group were excluded, leaving 968 eligible patients (484 with GI bleeding and 484 without GI bleeding). Four additional patients with GI bleeding were excluded because their ages did not meet the predefined age-stratum criteria. The final analytical cohort therefore comprised 964 patients, including 480 patients with GI bleeding and 484 patients without GI bleeding. Patients were subsequently stratified into four predefined age groups: preschool (3–6 years), school-age (7–12 years), adolescent (13–17 years), and adult (>18 years). A detailed participant flow diagram is provided in **Figure 1**.

**Figure 1:**
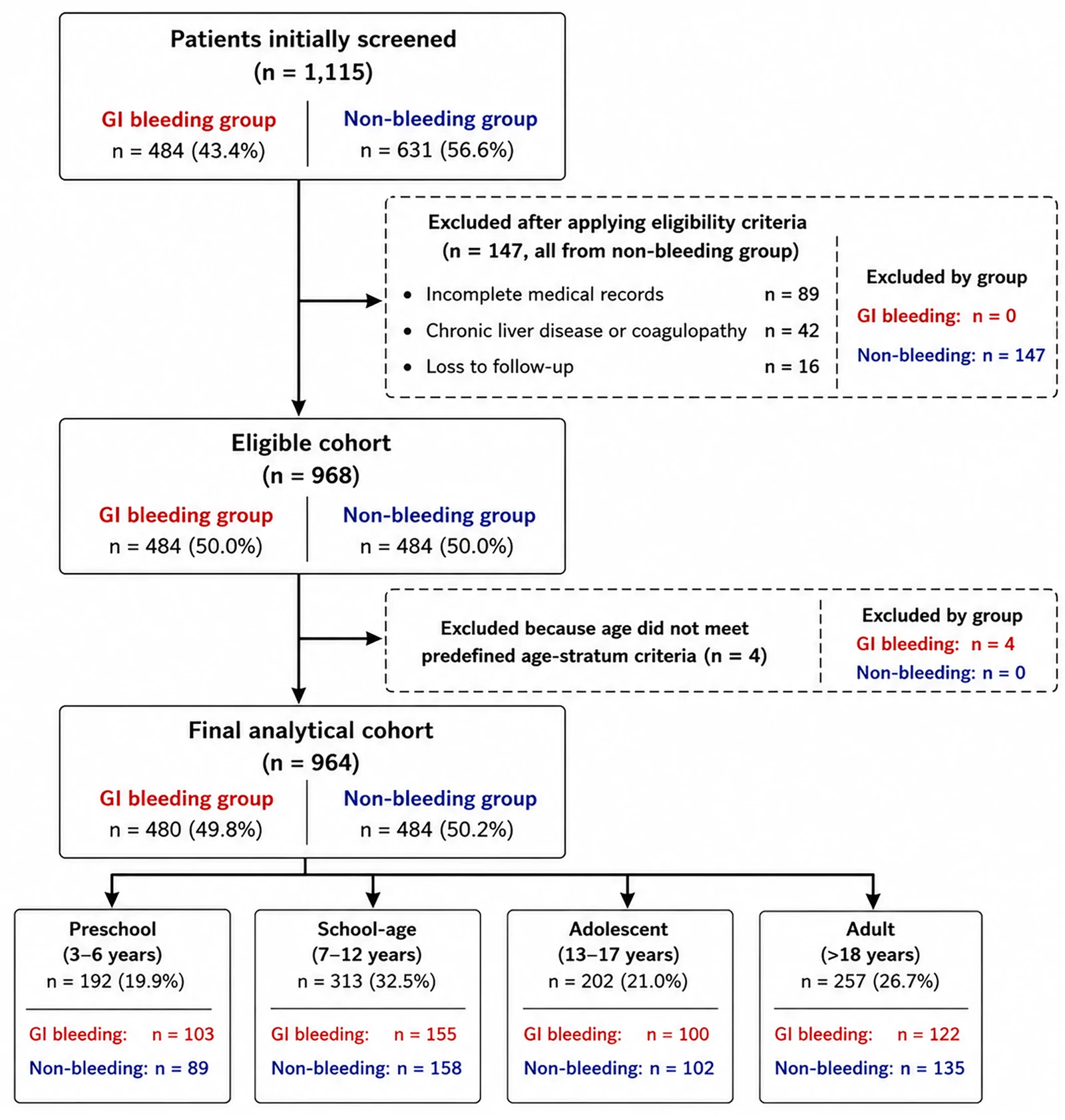
Flow diagram of patient selection and study cohort formation. A total of 1,115 patients with IgA vasculitis (IgAV) were initially screened, including 484 patients with gastrointestinal (GI) bleeding and 631 patients without GI bleeding. After application of the predefined eligibility criteria, 147 patients, all from the non-bleeding group, were excluded because of incomplete medical records (n=89), pre-existing chronic liver disease or coagulopathy (n=42), or loss to follow-up prior to outcome assessment (n=16), leaving 968 eligible patients (484 with GI bleeding and 484 without GI bleeding). Four additional patients, all from the GI bleeding group, were excluded because their ages did not meet the predefined age-stratum criteria. The final analytical cohort therefore consisted of 964 patients, including 480 patients with GI bleeding and 484 patients without GI bleeding. The cohort was subsequently stratified into preschool children aged 3–6 years (n=192; 103 bleeding and 89 non-bleeding), school-age children aged 7–12 years (n=313; 155 and 158), adolescents aged 13–17 years (n=202; 100 and 102), and adults aged >18 years (n=257; 122 and 135).

### Variable definitions and measurements

Age Groups: Patients were stratified into four cohorts for all subsequent analyses: Preschool (3–6 years), School-age (7–12 years), Adolescent (13–17 years), and Adult (>18 years). Season of Onset: Classified meteorologically: Spring (Mar-May), Summer (Jun-Aug), Autumn (Sep-Nov), Winter (Dec-Feb). For regression analysis, onset was dichotomized as Summer versus Other Seasons. Key Biomarker Calculations and Transformations: Neutrophil-to-Albumin Ratio (NAR): Calculated as absolute neutrophil count (×10^9^/L)/serum albumin (g/L). Given its right-skewed distribution, NAR was standardized as a z-score prior to inclusion in multivariable models. Fibrinogen-to-Globulin Ratio (F/G Ratio): Calculated as fibrinogen (g/L)/globulin (g/L). To approximate normality, a logarithmic transformation was applied, and the variable was entered into regression models as log(FIB/GLO). Systemic Immune-Inflammation Index (SII): Calculated as (Platelet count × Neutrophil count)/Lymphocyte count. SII values were categorized within each age stratum as Low (<25th percentile), Medium (25th–75th percentile), and High (>75th percentile). Mean Platelet Volume (MPV) was analyzed as a continuous z-score. Definition: GI bleeding was identified from documented clinical records and coded as a binary outcome in the final analytical dataset. Overt bleeding manifestations used for clinical ascertainment included hematemesis, melena, and hematochezia, and endoscopic evidence of gastrointestinal bleeding was also considered when available. Among the 480 patients classified as having GI bleeding, 135 (28.1%) had endoscopic confirmation. However, symptom-specific manifestations (hematemesis, melena, or hematochezia) were not retained as separate patient-level variables, and patient-level information linking endoscopic confirmation status and detailed endoscopic mucosal findings to the final analytical dataset was unavailable. Accordingly, age-specific differences in the prevalence or pattern of hematemesis, melena, hematochezia, and endoscopic mucosal lesions could not be evaluated in the present study.

### Laboratory tests and data collection

Demographic data, clinical features, and laboratory parameters recorded at the initial IgAV diagnosis were extracted from the hospital’s electronic medical records system. Key laboratory indices included: complete blood count (white blood cells, neutrophils, lymphocytes, platelets, MPV), coagulation profile (fibrinogen), and nutritional indices (serum albumin, globulin). All laboratory measurements were obtained at the time of diagnosis, prior to the initiation of treatment, and were performed in the hospital’s accredited central laboratory according to standardized procedures.

### Statistical analysis

All analyses were performed using R software. Descriptive statistics are presented as mean ± standard deviation (SD), median (IQR), or n (%), as appropriate. Comparisons between groups within each age stratum were conducted using the independent-samples t test, Mann–Whitney U test, or chi-square test, as appropriate. The primary analyses were conducted separately within each age stratum using Firth’s penalized logistic regression implemented with the logistf package in R. The models were fitted using Firth’s penalized maximum-likelihood estimation, and 95% confidence intervals and coefficient-level tests were based on the profile penalized likelihood (pl = TRUE). Firth’s penalized likelihood approach was used to reduce small-sample and sparse-data bias and to mitigate potential separation, particularly in the smaller age strata. Each age-specific model included summer onset, NAR (z-score), MPV (z-score), log(FIB/GLO), and SII category, with Low SII as the reference category. Results are reported as odds ratios (ORs) with 95% confidence intervals (CIs).

For each age-specific model, discrimination was evaluated using the area under the receiver operating characteristic curve (AUC). Model calibration was assessed using the Hosmer-Lemeshow goodness-of-fit test and the Brier score. Internal validation using 1,000 bootstrap resamples was performed to obtain optimism-corrected AUC estimates. This bootstrap procedure was used to assess optimism in model discrimination and was not used to evaluate the stability of the regression coefficients. All statistical tests were two-sided, and a P value <0.05 was considered statistically significant.

Given the multiple coefficient-level comparisons across the four age-stratified models, the Benjamini–Hochberg false discovery rate (FDR) procedure was applied to the 24 primary coefficient tests to account for multiplicity. Both nominal P values and FDR-adjusted q values are reported, with an FDR-adjusted q value <0.05 considered statistically significant after multiplicity correction. Because the age-stratified analyses were exploratory, differences in the patterns of associations across age groups were interpreted as hypothesis-generating rather than as formal evidence of differences in effect between age strata.

## Results

### Cohort assembly and baseline characteristics

A total of 1,115 patients with IgA vasculitis (IgAV) were initially identified, including 484 patients with gastrointestinal (GI) bleeding and 631 patients without. After exclusion of 147 patients, all from the non-bleeding group, according to the predefined eligibility criteria, 968 patients remained eligible (484 with GI bleeding and 484 without GI bleeding). Four additional patients, all from the GI bleeding group, were excluded because their ages did not meet the predefined age-stratum criteria, resulting in a final analytical cohort of 964 patients (GI bleeding group: n=480; non-bleeding group: n=484). The final cohort comprised 192 preschool children, 313 school-age children, 202 adolescents, and 257 adults. Baseline comparisons between bleeding and non-bleeding patients within each age stratum are presented in **Tables 1A**-**1D**. Consistent across all age groups, patients with GI bleeding had significantly higher levels of inflammatory markers, including white blood cell count, neutrophil count, and neutrophil-to-lymphocyte ratio (NLR), compared to controls (all P<0.01; **Tables 1A**-**1D**).

**Table 1A:** Comparison of laboratory indicators between patients in bleeding group and non-bleeding group(preschool).

Variable	Bleeding (N = 103)^1^	Non-bleeding (N = 89)^1^	P value^2^
Gender			0.8
Male	57 (55.3%)	52 (58.4%)	
Female	46 (44.7%)	37 (41.6%)	
Age (years)			0.001
3 years	6 (5.8%)	16 (18.0%)	
4 years	34 (33.0%)	11 (12.4%)	
5 years	24 (23.3%)	22 (24.7%)	
6 years	39 (37.9%)	40 (44.9%)	
WBC (×10^9/L)			0.003
Mean ± SD	14.01 ± 8.25	11.05 ± 4.17	
Median (Q1, Q3)	12.45 (9.16, 16.25)	10.59 (7.99, 12.80)	
NEU (×10^9/L)			<0.001
Mean ± SD	10.37 ± 7.90	7.12 ± 3.82	
Median (Q1, Q3)	8.40 (5.96, 12.62)	6.16 (4.53, 8.56)	
LYM (×10^9/L)			0.3
Mean ± SD	2.85 ± 1.40	3.05 ± 1.29	
Median (Q1, Q3)	2.53 (2.05, 3.21)	2.87 (2.38, 3.55)	
NLR			0.001
Mean ± SD	4.36 ± 3.81	2.83 ± 2.21	
Median (Q1, Q3)	3.22 (1.80, 5.46)	2.12 (1.44, 3.50)	
RBC (×10^12/L)			0.007
Mean ± SD	4.68 ± 0.37	4.54 ± 0.37	
Median (Q1, Q3)	4.70 (4.38, 4.95)	4.51 (4.29, 4.75)	
HGB (g/L)			0.014
Mean ± SD	129.48 ± 11.78	125.55 ± 9.92	
Median (Q1, Q3)	131.00 (122.00, 137.00)	124.00 (119.00, 133.00)	
PLT (×10^9/L)			0.7
Mean ± SD	396.88 ± 113.01	390.94 ± 130.05	
Median (Q1, Q3)	406.00 (331.00, 451.00)	368.00 (300.00, 455.00)	
PLR			0.4
Mean ± SD	159.91 ± 72.06	149.41 ± 96.49	
Median (Q1, Q3)	148.56 (115.28, 185.61)	126.36 (100.00, 167.39)	
MPV (fL)			0.2
Mean ± SD	8.87 ± 1.03	9.09 ± 1.17	
Median (Q1, Q3)	8.80 (8.20, 9.40)	9.10 (8.20, 9.80)	
PDW (%)			0.066
Mean ± SD	15.65 ± 0.66	15.37 ± 1.36	
Median (Q1, Q3)	15.70 (15.40, 15.90)	15.60 (15.40, 15.90)	
ALB (g/L)			0.002
Mean ± SD	40.15 ± 5.46	42.36 ± 3.88	
Median (Q1, Q3)	41.00 (37.60, 44.20)	41.90 (40.30, 45.70)	
GLO (g/L)			0.055
Mean ± SD	24.63 ± 4.52	25.90 ± 4.57	
Median (Q1, Q3)	24.20 (21.60, 27.60)	26.20 (22.70, 28.30)	
FIB (g/L)			0.2
Mean ± SD	3.42 ± 0.87	3.60 ± 1.06	
Median (Q1, Q3)	3.40 (2.90, 3.91)	3.43 (2.88, 4.10)	
A/G ratio			0.8
Mean ± SD	1.66 ± 0.28	1.68 ± 0.27	
Median (Q1, Q3)	1.67 (1.48, 1.86)	1.66 (1.52, 1.83)	
N/A ratio			0.003
Mean ± SD	0.29 ± 0.36	0.17 ± 0.09	
Median (Q1, Q3)	0.21 (0.14, 0.33)	0.15 (0.11, 0.20)	
F/G ratio			0.9
Mean ± SD	0.14 ± 0.04	0.14 ± 0.04	
Median (Q1, Q3)	0.14 (0.11, 0.17)	0.13 (0.12, 0.16)	
N*P/L ratio			0.004
Mean ± SD	1,841.81 ± 2,119.96	1,125.05 ± 952.77	
Median (Q1, Q3)	1,232.50(637.42,2,115.73)	780.94 (491.00, 1,391.70)	

^1^ n (%).

^2^ Fisher’s exact test; Two Sample t-test.

**Table 1B:** Comparison of laboratory indicators between patients in bleeding group and non-bleeding group(School-age).

Variable	Bleeding (N = 155)^1^	Non-bleeding (N = 158)^1^	P value^2^
Gender			0.2
Male	81 (52.3%)	94 (59.5%)	
Female	74 (47.7%)	64 (40.5%)	
Age (years)			0.4
7 years	35 (22.6%)	36 (22.8%)	
8 years	34 (21.9%)	34 (21.5%)	
9 years	33 (21.3%)	26 (16.5%)	
10 years	18 (11.6%)	23 (14.6%)	
11 years	19 (12.3%)	13 (8.2%)	
12 years	16 (10.3%)	26 (16.5%)	
WBC (×10^9/L)			<0.001
Mean ± SD	14.22 ± 7.38	10.18 ± 4.13	
Median (Q1, Q3)	12.72 (9.27, 16.86)	9.37 (7.46, 12.37)	
NEU (×10^9/L)			<0.001
Mean ± SD	11.09 ± 6.60	6.79 ± 3.87	
Median (Q1, Q3)	9.57 (6.78, 13.32)	6.04 (3.87, 8.58)	
LYM (×10^9/L)			0.005
Mean ± SD	2.26 ± 1.19	2.66 ± 1.25	
Median (Q1, Q3)	2.02 (1.50, 2.67)	2.52 (1.80, 3.24)	
NLR			<0.001
Mean ± SD	6.08 ± 4.77	3.47 ± 3.88	
Median (Q1, Q3)	4.60 (2.86, 8.05)	2.08 (1.47, 3.84)	
RBC (×10^12/L)			0.010
Mean ± SD	4.83 ± 0.45	4.70 ± 0.49	
Median (Q1, Q3)	4.77 (4.56, 5.06)	4.68 (4.34, 5.01)	
HGB (g/L)			0.002
Mean ± SD	135.88 ± 12.01	131.31 ± 13.56	
Median (Q1, Q3)	135.00(127.00,144.00)	131.00 (124.00, 141.00)	
PLT (×10^9/L)			<0.001
Mean ± SD	375.10 ± 105.07	332.37 ± 83.83	
Median (Q1, Q3)	358.00 (301.00, 446.00)	328.00 (270.00, 380.00)	
PLR			<0.001
Mean ± SD	200.64 ± 102.66	154.44 ± 92.68	
Median (Q1, Q3)	178.44 (122.91, 253.91)	129.31 (95.76, 183.86)	
MPV (fL)			0.10
Mean ± SD	9.31 ± 1.17	9.54 ± 1.29	
Median (Q1, Q3)	9.10 (8.50, 10.10)	9.30 (8.70, 10.30)	
PDW (%)			0.3
Mean ± SD	15.33 ± 1.76	15.13 ± 1.96	
Median (Q1, Q3)	15.80 (15.50, 16.00)	15.70 (15.50, 16.00)	
ALB (g/L)			<0.001
Mean ± SD	41.08 ± 4.99	42.91 ± 4.67	
Median (Q1, Q3)	42.10 (38.70, 44.60)	43.60 (40.10, 45.80)	
Min, Max	24.10, 50.40	16.20, 62.80	
GLO (g/L)			0.023
Mean ± SD	26.13 ± 4.69	27.31 ± 4.47	
Median (Q1, Q3)	26.80 (23.00, 29.00)	26.60 (24.20, 30.70)	
FIB (g/L)			0.057
Mean ± SD	3.61 ± 1.18	3.37 ± 1.06	
Median (Q1, Q3)	3.44 (2.74, 4.32)	3.20 (2.71, 3.97)	
A/G ratio			0.8
Mean ± SD	1.62 ± 0.34	1.61 ± 0.27	
Median (Q1, Q3)	1.58 (1.46, 1.72)	1.59 (1.42, 1.77)	
N/A ratio			<0.001
Mean ± SD	0.28 ± 0.20	0.16 ± 0.10	
Median (Q1, Q3)	0.23 (0.16, 0.33)	0.14 (0.09, 0.20)	
F/G ratio			0.003
Mean ± SD	0.14 ± 0.06	0.13 ± 0.04	
Median (Q1, Q3)	0.13 (0.10, 0.17)	0.12 (0.10, 0.15)	
N*P/L ratio			<0.001
Mean ± SD	2,345.63 ± 2,148.22	1,148.84 ± 1,258.73	
Median (Q1, Q3)	1,805.38 (1,001.22, 3,228.55)	694.34 (435.35, 1,362.32)	

^1^ n (%).

^2^ Fisher’s exact test; Two Sample t-test.

**Table 1C:** Comparison of laboratory indicators between patients in bleeding group and non-bleeding group(Adolescent).

Variable	Bleeding (N = 100)^1^	Non-bleeding (N = 102)^1^	P value^2^
Gender			0.085
Male	67 (67.0%)	56 (54.9%)	
Female	33 (33.0%)	46 (45.1%)	
Age (years)			0.9
13 years	22 (22.0%)	28 (27.5%)	
14 years	23 (23.0%)	25 (24.5%)	
15 years	24 (24.0%)	20 (19.6%)	
16 years	22 (22.0%)	20 (19.6%)	
17 years	9 (9.0%)	9 (8.8%)	
WBC (×10^9/L)			<0.001
Mean ± SD	14.62 ± 6.91	10.53 ± 4.55	
Median (Q1, Q3)	12.57 (9.72, 17.63)	9.40 (7.36, 12.87)	
NEU (×10^9/L)			<0.001
Mean ± SD	11.90 ± 6.65	7.42 ± 4.28	
Median (Q1, Q3)	10.69 (7.17, 15.62)	6.44 (4.56, 9.37)	
LYM (×10^9/L)			<0.001
Mean ± SD	1.83 ± 0.84	2.35 ± 1.18	
Median (Q1, Q3)	1.60 (1.28, 2.25)	2.00 (1.54, 3.04)	
NLR			<0.001
Mean ± SD	8.23 ± 6.48	3.99 ± 3.27	
Median (Q1, Q3)	6.70 (3.61, 10.38)	2.99 (1.86, 4.98)	
RBC (×10^12/L)			0.007
Mean ± SD	4.99 ± 0.61	4.78 ± 0.46	
Median (Q1, Q3)	5.00 (4.63, 5.35)	4.77 (4.45, 5.01)	
HGB (g/L)			0.010
Mean ± SD	145.28 ± 15.81	139.66 ± 14.71	
Median (Q1, Q3)	146.00 (134.00, 157.00)	140.00 (130.00, 150.00)	
PLT (×10^9/L)			<0.001
Mean ± SD	325.01 ± 79.39	287.02 ± 77.10	
Median (Q1, Q3)	318.00 (269.00, 367.50)	277.00 (231.00, 325.00)	
PLR			<0.001
Mean ± SD	216.42 ± 115.56	145.55 ± 66.15	
Median (Q1, Q3)	199.69 (135.47, 255.31)	138.82 (90.34, 185.00)	
MPV (fL)			<0.001
Mean ± SD	9.39 ± 1.12	10.02 ± 1.50	
Median (Q1, Q3)	9.15 (8.65, 10.15)	9.85 (9.10, 10.90)	
PDW (%)			0.036
Mean ± SD	15.65 ± 1.19	15.11 ± 2.29	
Median (Q1, Q3)	15.90 (15.60, 16.10)	15.90 (15.30, 16.20)	
ALB (g/L)			0.5
Mean ± SD	41.34 ± 5.82	41.84 ± 5.22	
Median (Q1, Q3)	41.65 (38.05, 46.35)	42.15 (39.40, 45.20)	
GLO (g/L)			0.2
Mean ± SD	26.34 ± 4.63	25.60 ± 4.46	
Median (Q1, Q3)	26.10 (22.70, 30.05)	25.05 (22.30, 28.10)	
FIB (g/L)			<0.001
Mean ± SD	4.03 ± 1.02	3.19 ± 1.03	
Median (Q1, Q3)	4.07 (3.23, 4.83)	2.98 (2.52, 3.78)	
A/G ratio			0.046
Mean ± SD	1.60 ± 0.24	1.67 ± 0.30	
Median (Q1, Q3)	1.58 (1.45, 1.72)	1.68 (1.52, 1.82)	
N/A ratio			<0.001
Mean ± SD	0.30 ± 0.21	0.19 ± 0.17	
Median (Q1, Q3)	0.25 (0.16, 0.39)	0.15 (0.11, 0.24)	
F/G ratio			<0.001
Mean ± SD	0.16 ± 0.05	0.13 ± 0.04	
Median (Q1, Q3)	0.15 (0.12, 0.19)	0.12 (0.10, 0.15)	
N*P/L ratio			<0.001
Mean ± SD	2,790.93 ± 2,490.84	1,180.16 ± 1,140.00	
Median (Q1, Q3)	2,138.59(1,133.66,3,418.53)	800.20(463.60,1,382.67)	

^1^ n (%).

^2^ Fisher’s exact test; Two Sample t-test.

**Table 1D:** Comparison of laboratory indicators between patients in bleeding group and non-bleeding group(Adult).

Variable	Bleeding (N = 122)^1^	Non-bleeding (N = 135)^1^	P value^2^
Gender			<0.001
Male	72 (59.0%)	43 (31.9%)	
Female	50 (41.0%)	92 (68.1%)	
Age (years)			0.10
Mean ± SD	44.16 ± 19.84	48.05 ± 17.64	
Median (Q1, Q3)	47.00 (22.00, 59.00)	50.00 (32.00, 61.00)	
WBC (×10^9/L)			<0.001
Mean ± SD	13.17 ± 6.51	9.23 ± 5.39	
Median (Q1, Q3)	12.28 (8.45, 16.82)	8.23 (6.54, 10.37)	
NEU (×10^9/L)			<0.001
Mean ± SD	10.70 ± 6.16	6.52 ± 5.02	
Median (Q1, Q3)	8.82 (6.19, 14.29)	5.22 (3.84, 7.90)	
LYM (×10^9/L)			0.001
Mean ± SD	1.64 ± 0.87	2.04 ± 1.06	
Median (Q1, Q3)	1.49 (1.04, 2.04)	1.79 (1.21, 2.54)	
NLR			<0.001
Mean ± SD	8.52 ± 8.06	4.13 ± 4.07	
Median (Q1, Q3)	6.64 (3.49, 10.33)	2.88 (1.80, 4.63)	
RBC (×10^12/L)			0.053
Mean ± SD	4.54 ± 0.79	4.36 ± 0.64	
Median (Q1, Q3)	4.68 (4.05, 5.07)	4.36 (3.97, 4.80)	
HGB (g/L)			0.077
Mean ± SD	134.30 ± 24.06	129.53 ± 18.89	
Median (Q1, Q3)	136.50 (117.00, 151.00)	131.00 (119.00, 143.00)	
PLT (×10^9/L)			<0.001
Mean ± SD	306.71 ± 116.04	253.96 ± 84.91	
Median (Q1, Q3)	298.00 (229.00, 367.00)	237.00 (205.00, 289.00)	
PLR			<0.001
Mean ± SD	228.25 ± 136.10	152.05 ± 92.12	
Median (Q1, Q3)	198.89 (140.16, 273.47)	131.41 (101.92, 182.91)	
MPV (fL)			<0.001
Mean ± SD	9.61 ± 1.25	10.28 ± 1.49	
Median (Q1, Q3)	9.50 (8.70, 10.50)	10.20 (9.20, 11.10)	
Min, Max	7.50, 13.40	6.90, 15.20	
PDW (%)			0.8
Mean ± SD	15.04 ± 2.24	14.96 ± 2.27	
Median (Q1, Q3)	15.90 (15.40, 16.30)	15.80 (13.50, 16.40)	
ALB (g/L)			0.005
Mean ± SD	37.46 ± 6.71	39.53 ± 5.02	
Median (Q1, Q3)	38.25 (31.70, 42.20)	39.90 (36.70, 42.40)	
GLO (g/L)			0.003
Mean ± SD	26.17 ± 5.39	28.23 ± 5.61	
Median (Q1, Q3)	26.50 (22.70, 28.80)	27.60 (23.90, 32.10)	
FIB (g/L)			0.007
Mean ± SD	4.25 ± 1.30	3.81 ± 1.31	
Median (Q1, Q3)	4.11 (3.36, 5.28)	3.47 (2.94, 4.60)	
A/G ratio			0.6
Mean ± SD	1.46 ± 0.27	1.45 ± 0.30	
Median (Q1, Q3)	1.47 (1.32, 1.61)	1.44 (1.22, 1.64)	
N/A ratio			<0.001
Mean ± SD	0.30 ± 0.21	0.17 ± 0.16	
Median (Q1, Q3)	0.24 (0.16, 0.38)	0.13 (0.09, 0.20)	
F/G ratio			<0.001
Mean ± SD	0.17 ± 0.06	0.14 ± 0.05	
Median (Q1, Q3)	0.16 (0.12, 0.20)	0.13 (0.10, 0.16)	
N*P/L ratio			<0.001
Mean ± SD	2,690.20 ± 2,761.18	1,067.71 ± 1,315.99	
Median (Q1, Q3)	1,799.61(941.82,3,471.23)	728.69 (447.40, 1,137.98)	

^1^ n (%).

^2^ Fisher’s exact test; Two Sample t-test.

WBC, White Blood Cell Count; NEU, Neutrophil Count; LYM, Lymphocyte Count; NLR, Neutrophil-to-Lymphocyte Ratio; RBC, Red Blood Cell Count; HGB, Hemoglobin; PLT, Platelet Count; PLR, Platelet-to-Lymphocyte Ratio; MPV, Mean Platelet Volume; PDW, Platelet Distribution Width; ALB, Albumin; GLO, Globulin; FIB, Fibrinogen; A/G ratio, Albumin-to-Globulin ratio; NEU/ALB ratio:Neutrophil-to-Albumin ratio; FIB/GLO ratio, Fibrinogen-to-Globulin ratio; NEUPLT/LYM ratio, Neutrophil × Platelet/Lymphocyte ratio.

### Age-stratified multivariable analysis of risk factors

Age-stratified Firth penalized logistic regression models were fitted to evaluate factors associated with gastrointestinal (GI) bleeding (**Table 2**). The pattern of factors associated with GI bleeding differed across the four age strata. In the preschool group (3–6 years), an elevated neutrophil-to-albumin ratio (NAR) was significantly associated with GI bleeding in the Firth penalized model (odds ratio [OR], 5.88; 95% confidence interval [CI], 1.90–21.37; P = 0.001). Although the relatively wide confidence interval indicates limited precision of the effect-size estimate, the association persisted under penalized likelihood estimation, which reduces small-sample and sparse-data bias. For school-age children (7–12 years), the risk factors were more complex. A higher NAR remained significant (OR = 2.02, 95% CI: 1.27–3.45, P = 0.002). Additionally, summer onset of the disease (OR = 2.47, 95% CI: 1.19–5.33, P = 0.015) and a medium (OR = 2.72, 95% CI: 1.37–5.58, P = 0.004) or high (OR = 3.70, 95% CI: 1.36–10.25, P = 0.011) SII level were independent predictors. The seasonal distribution and the proportions of summer onset across the four age groups are shown in **Figures 2**, **3**. In the adolescent group (13–17 years), the pattern shifted. The NAR was no longer a significant predictor (P = 0.986). Instead, the model highlighted the role of systemic inflammation and coagulation. A high SII (OR = 6.22, 95% CI: 1.86–21.42, P = 0.003) and a higher log-transformed FIB/GLO ratio (OR = 4.57, 95% CI: 1.55–14.37, P = 0.005) were independent risk factors. A lower MPV showed a nominal association with increased odds of GI bleeding (OR = 0.68, 95% CI: 0.47–0.95, P = 0.025), although this association did not remain statistically significant after FDR correction (q=0.062). The model for adults (>18 years) was characterized by the predominant role of systemic inflammation. A high SII was the strongest independent risk factor (OR = 9.53, 95% CI: 3.00–31.58, P<0.001, q=0.003). Medium SII showed a nominal association (OR = 2.27, 95% CI: 1.10–4.87, P = 0.026), but this association did not remain statistically significant after FDR correction (q=0.062). No other variables, including NAR or coagulation markers, retained independent significance in this age group. After correction for multiple comparisons using the Benjamini–Hochberg FDR procedure across the 24 coefficient-level tests, the principal associations remained statistically significant for NAR in preschool children (q=0.013); NAR (q=0.014), summer onset (q=0.044), medium SII (q=0.019), and high SII (q=0.036) in school-age children; high SII (q=0.019) and log(FIB/GLO) (q=0.022) in adolescents; and high SII (q=0.003) in adults. In contrast, the nominal associations of lower MPV in adolescents (P = 0.025, q=0.062) and medium SII in adults (P = 0.026, q=0.062) did not remain statistically significant after FDR correction. Accordingly, the observed differences in association patterns across age strata were interpreted as exploratory rather than as formal evidence of age-dependent differences in effect. The age-stratified regression results are illustrated in **Figures 4**, **5**

**Table 2A:** Firth penalized logistic regression analysis of factors associated with GI bleeding in IgAV patients (preschool).

Variable	OR	95% CI	P-value	FDR-adjusted q value	Significant after FDR correction
Summer onset (vs Other seasons)	1.45	0.60-3.66	0.414	0.585	No
Neutrophil/Albumin ratio (z-score)	5.88	1.90-21.37	0.001	0.013	Yes
Mean platelet volume (z-score)	0.95	0.70-1.28	0.718	0.783	No
Log(Fibrinogen/Globulin ratio)	0.69	0.23-2.01	0.502	0.659	No
Medium SII (vs Low)	0.77	0.35-1.69	0.521	0.659	No
High SII (vs Low)	0.51	0.14-1.72	0.279	0.418	No

SII, Systemic immune-inflammation index.

ORs and 95% CIs were estimated using Firth’s penalized logistic regression. SII, systemic immune-inflammation index; NAR, neutrophil-to-albumin ratio; MPV, mean platelet volume.

P values were adjusted for multiple comparisons across the 24 coefficient-level tests in the four age-stratified models using the Benjamini–Hochberg false discovery rate (FDR) procedure. An FDR-adjusted q value <0.05 was considered statistically significant after correction for multiple comparisons.

**Table 2B:** Firth penalized logistic regression analysis of factors associated with GI bleeding in IgAV patients (School-age).

Variable	OR	95% CI	P-value	FDR-adjusted q value	Significant after FDR correction
Summer onset (vs Other seasons)	2.47	1.19-5.33	0.015	0.044	Yes
Neutrophil/Albumin ratio (z-score)	2.02	1.27-3.45	0.002	0.014	Yes
Mean platelet volume (z-score)	0.96	0.75-1.23	0.763	0.796	No
Log(Fibrinogen/Globulin ratio)	2.09	0.96-4.72	0.065	0.117	No
Medium SII (vs Low)	2.72	1.37-5.58	0.004	0.019	Yes
High SII (vs Low)	3.70	1.36-10.25	0.011	0.036	Yes

SII, Systemic immune-inflammation index.

ORs and 95% CIs were estimated using Firth’s penalized logistic regression. SII, systemic immune-inflammation index; NAR, neutrophil-to-albumin ratio; MPV, mean platelet volume.

P values were adjusted for multiple comparisons across the 24 coefficient-level tests in the four age-stratified models using the Benjamini–Hochberg false discovery rate (FDR) procedure. An FDR-adjusted q value <0.05 was considered statistically significant after correction for multiple comparisons.

**Table 2C:** Firth penalized logistic regression analysis of factors associated with GI bleeding in IgAV patients (Adolescent).

Variable	OR	95% CI	P-value	FDR-adjusted q value	Significant after FDR correction
Summer onset (vs Other seasons)	2.30	0.94-5.90	0.068	0.117	No
Neutrophil/Albumin ratio (z-score)	1.00	0.65-1.79	0.986	0.986	No
Mean platelet volume (z-score)	0.68	0.47-0.95	0.025	0.062	No
Log(Fibrinogen/Globulin ratio)	4.57	1.55-14.37	0.005	0.022	Yes
Medium SII (vs Low)	1.81	0.81-4.19	0.149	0.238	No
High SII (vs Low)	6.22	1.86-21.42	0.003	0.019	Yes

SII, Systemic immune-inflammation index.

ORs and 95% CIs were estimated using Firth’s penalized logistic regression. SII, systemic immune-inflammation index; NAR, neutrophil-to-albumin ratio; MPV, mean platelet volume.

P values were adjusted for multiple comparisons across the 24 coefficient-level tests in the four age-stratified models using the Benjamini–Hochberg false discovery rate (FDR) procedure. An FDR-adjusted q value <0.05 was considered statistically significant after correction for multiple comparisons.

**Table 2D:** Firth penalized logistic regression analysis of factors associated with GI bleeding in IgAV patients (Adult).

Variable	OR	95% CI	P-value	FDR-adjusted q value	Significant after FDR correction
Summer onset (vs Other seasons)	1.14	0.57-2.25	0.710	0.783	No
Neutrophil/Albumin ratio (z-score)	1.11	0.74-1.88	0.640	0.768	No
Mean platelet volume (z-score)	0.76	0.57-1.02	0.066	0.117	No
Log(Fibrinogen/Globulin ratio)	2.09	0.96-4.67	0.064	0.117	No
Medium SII (vs Low)	2.27	1.10-4.87	0.026	0.062	No
High SII (vs Low)	9.53	3.00-31.58	<0.001	0.003	Yes

SII, Systemic immune-inflammation index.

ORs and 95% CIs were estimated using Firth’s penalized logistic regression. SII, systemic immune-inflammation index; NAR, neutrophil-to-albumin ratio; MPV, mean platelet volume.

P values were adjusted for multiple comparisons across the 24 coefficient-level tests in the four age-stratified models using the Benjamini–Hochberg false discovery rate (FDR) procedure. An FDR-adjusted q value <0.05 was considered statistically significant after correction for multiple comparisons.

**Figure 2:**
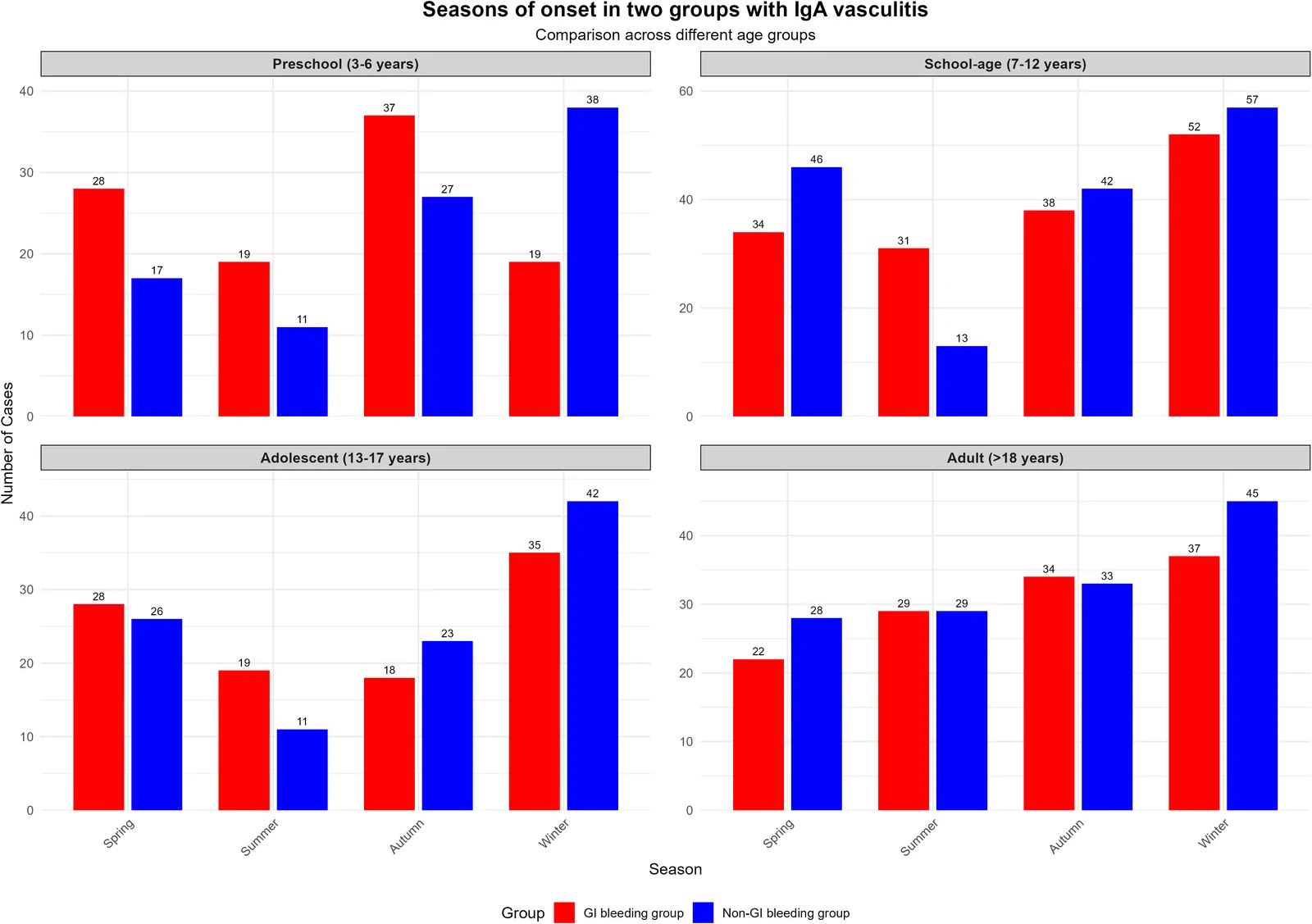
Seasonal distribution of IgA vasculitis (IgAV) patients across different age groups. The four-panel figure presents the number of patients with disease onset in each season (Spring, Summer, Autumn, Winter), comparing the GI bleeding group to the non-bleeding group. Each panel corresponds to one age cohort: A, Preschool (3–6 years); B, School-age (7–12 years); C, Adolescent (13–17 years); D, Adult (>18 years). Consistent with the multivariable regression results, a visual predominance of summer onset in the GI bleeding group is notably apparent in the school-age cohort, the only age group where summer onset was identified as an independent risk factor.

**Figure 3:**
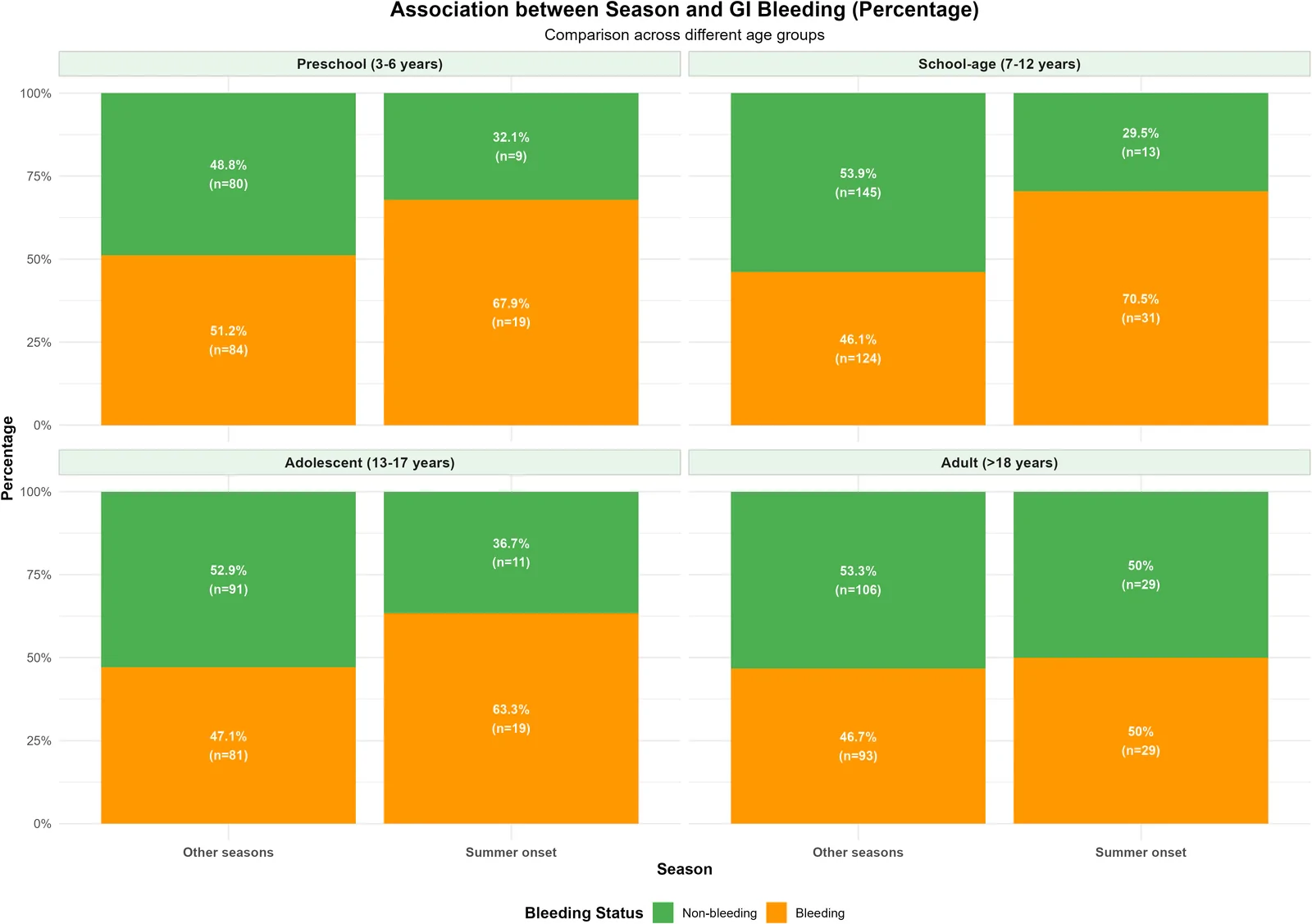
Association between summer onset and gastrointestinal (GI) bleeding across age groups. The four-panel figure compares the proportion of patients with summer onset versus other-season onset between the GI bleeding and non-bleeding groups. Each panel represents one age cohort: A, Preschool (3–6 years); B, School-age (7–12 years); C, Adolescent (13–17 years); D, Adult (>18 years). Data are presented as percentages within each bleeding status group. This visualization directly illustrates the finding from multivariable analysis, where summer onset emerged as a statistically significant independent risk factor exclusively in the school-age cohort.

**Figure 4:**
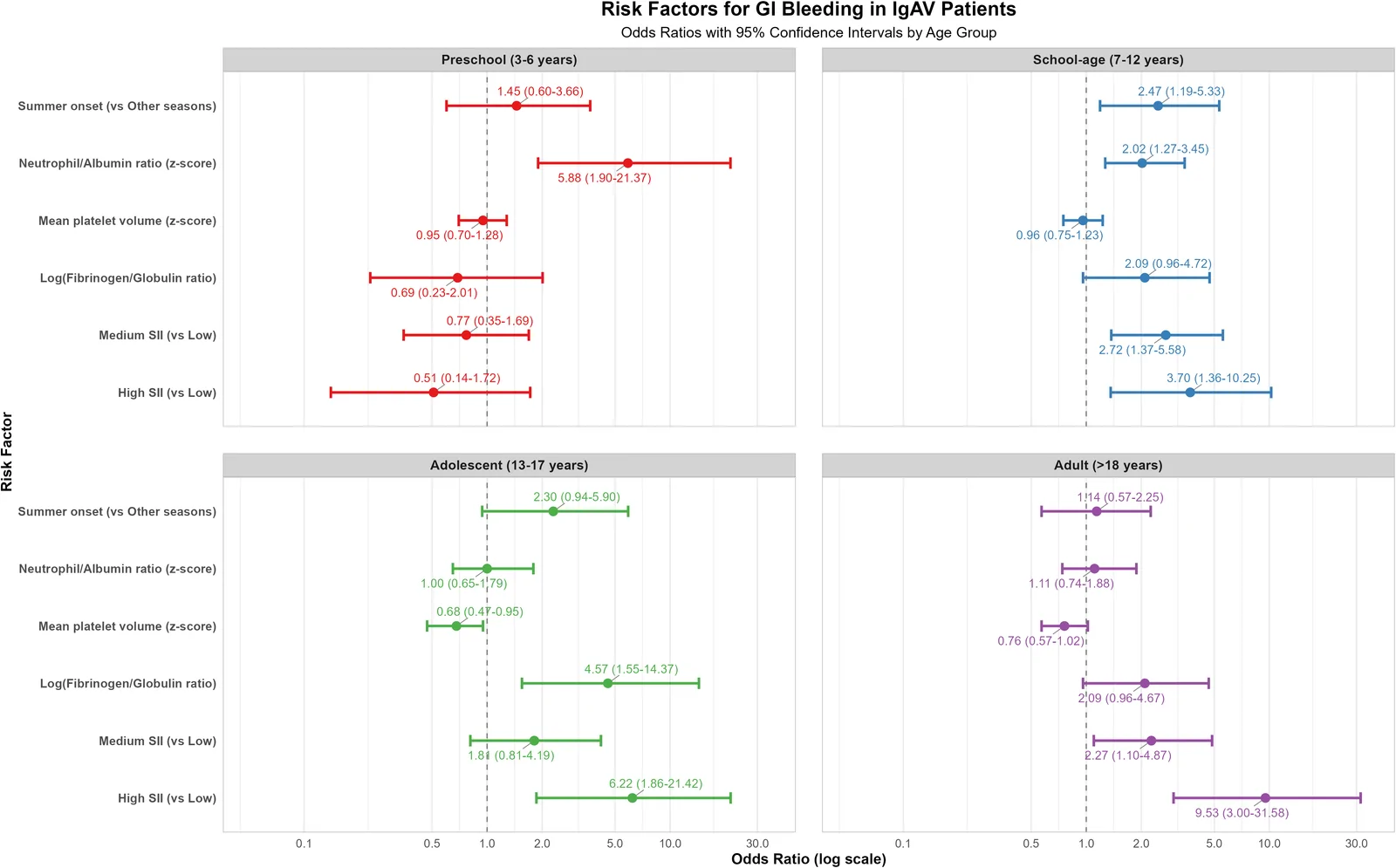
Independent risk factors for gastrointestinal bleeding in different age groups with IgA vasculitis. Forest plots display odds ratios (ORs) with 95% confidence intervals from the age-specific Firth penalized logistic regression models. Associations remaining statistically significant after Benjamini–Hochberg FDR correction included NAR in preschool children; NAR, summer onset, and medium/high SII in school-age children; high SII and log(FIB/GLO) in adolescents; and high SII in adults.

**Figure 5:**
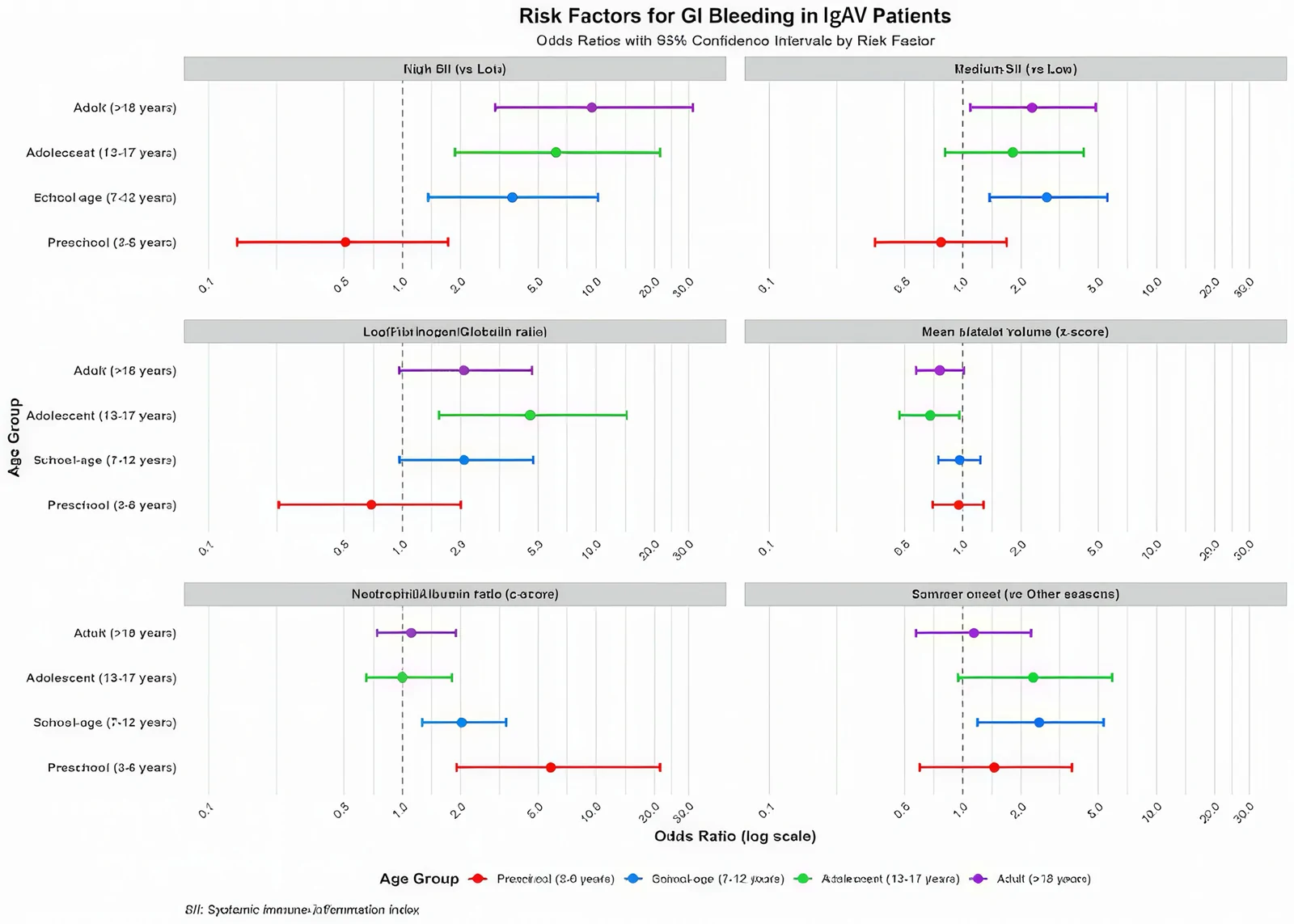
Forest plots of age-stratified Firth penalized logistic regression analyses for factors associated with gastrointestinal bleeding in IgA vasculitis. The figure illustrates the heterogeneous patterns of association observed across age strata: an elevated neutrophil-to-albumin ratio (NAR) was the sole significant factor in preschool children (OR = 5.88), while school-age children exhibited additional risks from summer onset (OR = 2.47) and medium/high systemic immune-inflammation index (SII). In adolescents, the risk pattern shifted toward high SII (OR = 6.22) and an elevated log-transformed fibrinogen-to-globulin ratio (OR = 4.57), with lower mean platelet volume showing only a nominal association that did not remain statistically significant after FDR correction. In adults, high SII showed the strongest association (OR = 9.53). Horizontal bars indicate 95% confidence intervals; the vertical dashed line marks the null effect (OR = 1). Associations meeting the FDR-adjusted significance threshold (q<0.05) are identified as statistically significant after correction for multiple comparisons.

### Predictive performance of the models

The discriminative ability of each age-specific prediction model was assessed using the area under the receiver operating characteristic curve (AUC). Following internal validation with 1,000 bootstrap samples to correct for optimism, the AUC values were as follows: 0.686 (95% CI: 0.611-0.761) for the preschool model, 0.763 (95% CI: 0.711-0.816) for the school-age model, 0.771 (95% CI: 0.707-0.835) for the adolescent model, and 0.784 (95% CI: 0.726-0.841) for the adult model. Overall, model discrimination improved progressively with increasing age. Calibration was acceptable across all models, as indicated by non-significant Hosmer–Lemeshow tests (P>0.05). The ROC curves for the age-specific models and individual candidate factors are shown in **Figures 6**, **7**, respectively.

**Figure 6:**
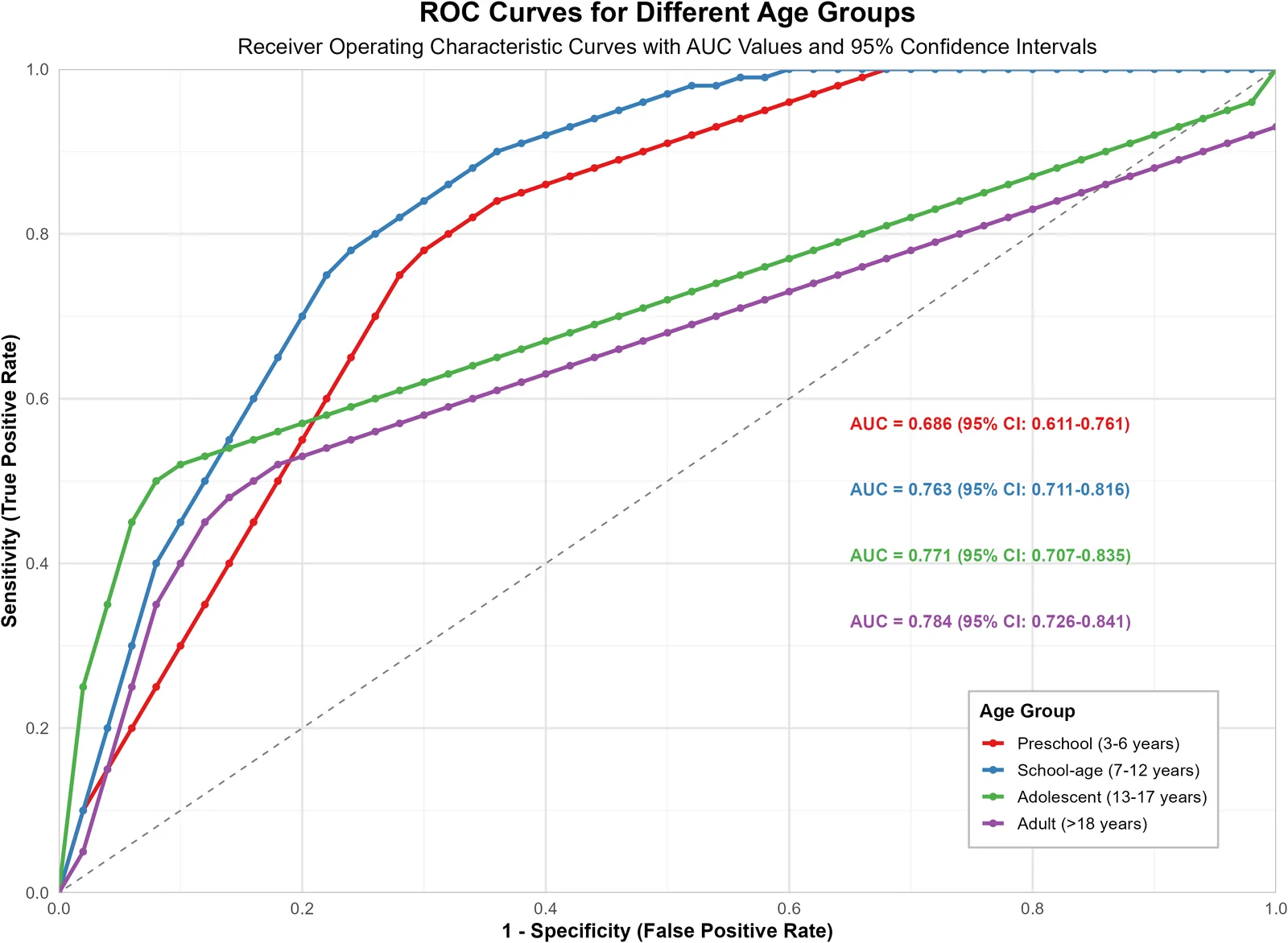
Receiver operating characteristic (ROC) curves for the age-stratified prediction models of gastrointestinal bleeding in IgA vasculitis. The figure shows the discriminative performance of each age-specific Firth penalized logistic regression model. The area under the curve (AUC) with 95% confidence interval for each model, after optimism correction via bootstrapping, was: preschool (3–6 years): 0.686 (0.611-0.761); school-age (7–12 years): 0.763 (0.711-0.816); adolescent (13–17 years): 0.771 (0.707-0.835); adult (>18 years): 0.784 (0.726-0.841). The diagonal dashed line indicates a performance no better than random chance (AUC = 0.5).

**Figure 7:**
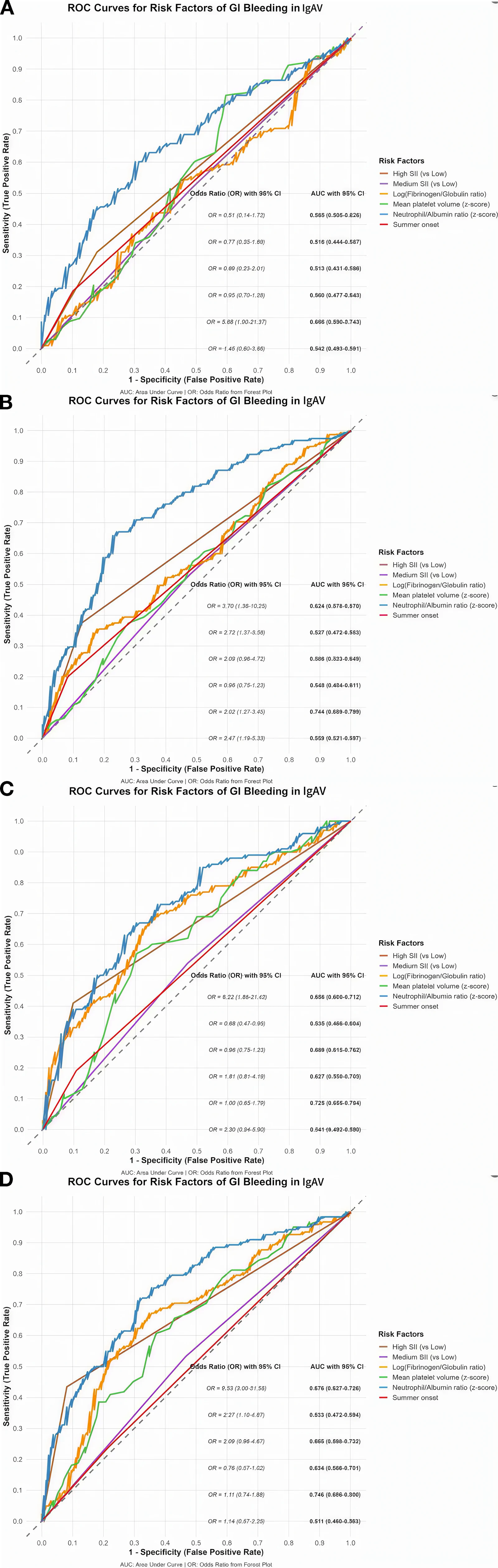
**(A)** Predictive performance of individual risk factors for GI bleeding in preschool children (3–6 years) with IgA vasculitis. The figure presents the receiver operating characteristic (ROC) curves for each candidate variable. The corresponding area under the curve (AUC) with 95% confidence interval and odds ratio (OR) with 95% CI are listed. The neutrophil-to-albumin ratio (NAR) showed the highest discriminative ability (AUC = 0.666), while other factors (e.g., SII, fibrinogen-to-globulin ratio) performed near chance (AUC≈0.5). The dashed line indicates no discrimination (AUC = 0.5). **(B)** Discriminative performance of individual candidate risk factors for gastrointestinal bleeding in school-age children (7–12 years) with IgA vasculitis. Receiver operating characteristic (ROC) curves illustrate the univariable discriminative ability of each candidate variable. The corresponding AUC with 95% confidence interval **(CI)** are shown together with adjusted ORs (95% CIs) from the age-specific multivariable Firth model. The neutrophil-to-albumin ratio (NAR) demonstrated the highest discriminative power (AUC = 0.744, 95% CI: 0.689–0.799; OR = 2.02, 95% CI: 1.27–3.45), followed by high systemic immune-inflammation index (SII) (AUC = 0.624). The remaining factors showed limited discriminatory capacity (AUC close to 0.5). The diagonal dashed line indicates no discrimination (AUC = 0.5). **(C)** Discriminative performance of individual risk factors for GI bleeding in adolescents (13–17 years) with IgA vasculitis. Among the individual candidate variables, NAR showed the highest AUC (0.725), indicating the greatest univariable discrimination in this age group. However, NAR did not retain an independent association in the multivariable Firth model (OR = 1.00, P = 0.986). Log(FIB/GLO) and high SII had AUCs of 0.689 and 0.656, respectively, and both remained independently associated with GI bleeding. **(D)** Discriminative performance of individual risk factors for GI bleeding in adult patients (>18 years) with IgA vasculitis. NAR showed the highest individual AUC (0.746) in adults, whereas high SII had an AUC of 0.676. Despite its lower univariable discriminative performance, high SII emerged as the strongest independently associated factor in the multivariable Firth model (OR = 9.53), whereas NAR did not retain independent significance.

## Discussion

Principal Findings: This age-stratified study identified heterogeneous patterns of factors associated with gastrointestinal (GI) bleeding in patients with IgA vasculitis (IgAV). NAR showed a prominent association in preschool children and remained associated with GI bleeding in school-age children, whereas SII and coagulation-related markers were more prominent in older age groups. Importantly, most of the principal associations remained statistically significant after FDR correction for multiple comparisons, although the nominal associations of lower MPV in adolescents and medium SII in adults were attenuated after correction. Because the analyses were stratified and exploratory, these patterns should be interpreted as age-related heterogeneity in observed associations rather than as definitive evidence of an age-dependent biological progression. In early childhood (3–6 years), the observed association pattern was dominated by the neutrophil-to-albumin ratio (NAR), which integrates neutrophil burden and serum albumin status. During school age (7–12 years), the risk profile broadens to encompass systemic immune inflammation (SII) together with a distinct seasonal association, particularly during summer (13, 14). In adolescents (13–17 years), the observed association pattern was characterized by high SII and an elevated fibrinogen-to-globulin ratio, whereas NAR was not independently associated with GI bleeding. Finally, in adulthood (>18 years), the high SII showed the strongest association. Numerically, the optimism-corrected AUC values ranged from 0.686 in preschool children to 0.784 in adults; however, formal between-group comparisons of AUCs were not performed.

Comparison with Existing Literature: Our findings contextualize and clarify inconsistencies in previous research. Many prior studies analyzing IgAV cohorts as a whole reported heterogeneous associations for individual inflammatory markers, such as neutrophil count or NLR (15, 16). Our findings suggest that NAR was more strongly associated with GI bleeding in younger children, whereas SII showed stronger independent associations in adolescents and adults. Although both NAR and SII are influenced by neutrophil-related inflammation, they capture different biological dimensions. NAR reflects the balance between neutrophil burden and serum albumin (17), whereas SII integrates neutrophil, lymphocyte, and platelet counts and may therefore provide a broader representation of systemic immune-inflammatory activity (15, 16). In adolescents and adults, systemic inflammation may involve coordinated changes in neutrophils, lymphocytes, and platelets that are more effectively captured by SII, while albumin levels may be influenced by additional factors such as nutritional status, hepatic synthesis, vascular permeability, and chronic comorbid conditions. Moreover, the lack of an independent association for NAR in the multivariable models does not imply an absence of inflammation; rather, it suggests that NAR may provide limited additional discriminatory information once broader inflammatory indices such as SII are considered. This may partly explain why SII, but not NAR, remained independently associated with GI bleeding in adolescents and adults. The isolated association of summer onset with bleeding risk exclusively in school-age children aligns with epidemiological studies highlighting a higher incidence of antecedent infections in this active demographic and underscores that environmental triggers may have age-windowed effects. The strong association of high SII with GI bleeding in adults is consistent with literature characterizing adult-onset IgAV as a more severe, systemically inflamed phenotype with distinct outcomes (9, 12). The novel identification of the fibrinogen-to-globulin ratio as an independent risk factor in adolescents introduces a previously underappreciated dimension of coagulation dysregulation into the pathophysiology of IgAV-related bleeding during this developmental stage.

Interpretation and Proposed Mechanisms: The observed age-stratified patterns may partly reflect fundamental shifts in host-pathogen interaction and vascular pathophysiology across developmental stages. From a developmental perspective, substantial physiological and immunological changes occur across childhood, adolescence, and adulthood. In preschool children, both innate and adaptive immunity remain under active maturation, with age-dependent differences in inflammatory responsiveness and the establishment of immunological memory. During school age, increasing immune maturation and cumulative antigen exposure contribute to more coordinated innate–adaptive immune responses, whereas adolescence is accompanied by further maturation of immune regulation and the hemostatic system, with coagulation and anticoagulant factors progressively approaching adult patterns. By adulthood, immune and hemostatic regulation is largely mature, while systemic inflammatory responses may increasingly reflect cumulative environmental exposures and comorbid conditions. The prominence of NAR in preschool children may reflect relatively greater neutrophil-predominant inflammatory activity at this developmental stage (18), with hypoalbuminemia potentially indicating significant vascular leakage (19). The additional associations observed in school-age children may be related to the immune response, characterized by the emergence of a more integrated systemic inflammatory component together with increased susceptibility to common environmental triggers, such as summer-associated pathogens.

The transition observed during adolescence, characterized by the emergence of coagulation-related markers alongside elevated SII, may indicate increasing involvement of the inflammation–coagulation interface (20). Systemic inflammation at this stage may be accompanied by alterations in endothelial and coagulation-related pathways (21, 22). These combined inflammatory and coagulation-related changes may contribute to impaired vascular integrity and altered hemostatic balance. The association between lower mean platelet volume (MPV) and bleeding risk in this group may further reflect platelet activation or consumption (23). In adults, the strong association of high SII with GI bleeding may reflect a greater contribution of systemic inflammatory activity in the context of the more severe clinical phenotype and greater comorbidity burden observed in adult IgAV (24).

Summer Onset and Potential Infectious Triggers: The exclusive association of summer onset with GI bleeding in the school-age cohort warrants particular attention. Importantly, our finding reflects a higher likelihood of GI bleeding among school-age patients with IgAV presenting in summer, rather than a higher overall incidence of IgAV during summer. Previous epidemiological studies have demonstrated seasonal variation in pediatric IgAV and suggested an important contribution of seasonally circulating infectious pathogens (13, 14). Thus, summer onset may partly act as a proxy for differences in infectious exposure or other season-related environmental factors rather than representing a direct seasonal effect. In particular, antecedent infection may represent an unmeasured confounder, because recent enteric or respiratory infections could influence both the timing of IgAV onset and the risk of gastrointestinal involvement. However, our retrospective dataset did not systematically capture the type, timing, or microbiological confirmation of preceding infections. Therefore, we cannot determine whether the observed summer association reflects a direct seasonal effect or differences in antecedent infectious exposure. This possibility remains speculative and warrants future prospective studies incorporating standardized infection history and pathogen surveillance. However, our study did not include meteorological variables, such as ambient temperature, humidity, or extreme-heat exposure, nor systematic pathogen surveillance. Therefore, we cannot determine whether climate warming contributed to the observed summer association. This possibility remains speculative and warrants future studies integrating meteorological and infection-surveillance data.

Limitations: Several limitations of this study merit consideration. First, the retrospective, single-center design inherently constrains causal inference and may limit the generalizability of our findings; external validation in prospective, multiethnic cohorts is therefore warranted. Second, despite age-stratified analyses, residual confounding related to demographic characteristics and other unmeasured or unrecorded variables (e.g., detailed medication history prior to presentation, genetic factors, or nuanced clinical severity scores) cannot be excluded. Third, the diagnosis of GI bleeding relied on clinical criteria for a majority of patients, though this reflects real-world practice where endoscopy is not always feasible (25). Although 135 of 480 patients with GI bleeding had endoscopic confirmation, patient-level information linking endoscopic confirmation status to the final analytical dataset was not retained. In addition, specific bleeding manifestations, including hematemesis, melena, and hematochezia, as well as detailed endoscopic mucosal findings, were not preserved as separate patient-level variables. Consequently, we were unable to compare the prevalence or pattern of clinical bleeding manifestations and endoscopic lesions across age strata or to perform a sensitivity analysis comparing endoscopically confirmed cases with clinically diagnosed cases. This limitation introduces potential heterogeneity in outcome ascertainment and prevents us from determining whether the observed associations differ according to bleeding phenotype or method of confirmation. Future prospective studies should incorporate standardized recording of bleeding manifestations and endoscopic findings. Finally, the mechanistic explanations proposed for the observed age-related trends, while biologically plausible and supported by our data, remain hypothesis-generating and require confirmation in future studies incorporating direct assessments of endothelial function, cytokine profiles, and platelet activity (26, 27). Although Firth’s penalized logistic regression was used to reduce small-sample bias, the relatively wide confidence interval for NAR in the preschool group indicates limited precision of the effect-size estimate; this finding therefore warrants cautious interpretation and external validation.

## Conclusion

In conclusion, the factors associated with GI bleeding in IgAV showed heterogeneous patterns across age strata, ranging from predominantly neutrophil-related inflammatory associations in younger children to broader systemic inflammatory and coagulation-related associations in older groups. Most principal associations remained statistically significant after FDR correction for multiple comparisons. Nevertheless, because the age-stratified analyses were exploratory and formal age-by-predictor interactions were not evaluated, these findings should be regarded as hypothesis-generating. Prospective studies are needed to validate these age-specific patterns and determine whether they translate into clinically meaningful differences in risk assessment.

## Data Availability

The datasets used in this study are derived from the electronic medical records of the Affiliated Hospital of Southwest Medical University. Due to patient privacy protection requirements and institutional data management policies, the raw data cannot be publicly shared or included in the article/supplementary materials. However, access to de-identified data can be requested from the corresponding author upon reasonable request, subject to approval from the hospital ethics committee.
